# Supplementary material for: Micro/Nanostructured Coating for Cotton Textiles That Repel Oil, Water, and Chemical Warfare Agents
Source: Polymers (Basel). 2020 Aug 14;12(8):1826. doi: 10.3390/polym12081826 (PMC7464014; doi:10.3390/polym12081826)
Supplement: Supplementary file 1 [file polymers-12-01826-s001.pdf]

Supporting Information

Micro/Nanostructured Coating for Cotton Textiles that Repel Oil, Water, and Chemical Warfare Agents

*Jihyun Kwon, Hyunsook Jung, Heesoo Jung, Juno Lee**.

Agency for Defense Development, Yuseong-Gu, Daejeon 34186, South Korea

E-mail: juno@add.re.kr

This supplementary information includes the following sections:

Table S1: TGA and DTG data

Figure S1: Surface for height of small structure.

Figure S2: SEM image of OmniBlock coated cotton fabric.

Figure S3: The optical images of water and n-dodecane droplet on the cotton fabric.

Figure S4: Optical image of HD and GD droplets.

Figure S5: Optical images of shedding angle for n-dodecane

Figure S6: The optical images of knotted cotton fabric.

Table S1. TGA and DTG data of raw and OmniBlock coated cotton fabrics in air and nitrogen.

Atmosphere	Sample	$T_{5\%}$ (℃)	$T_{10\%}$ (℃)	T_{max1} (℃)	Residue at T_{max1} (%)	T_{max2} (℃)	Residue at T_{max2} (%)	Residue at 800 ℃(%)
Air	Raw cotton	90	260	327	45.6	415	7.6	1.3
	OmniBlock coated cotton	192	273	324	58.2	426	19.4	9.6
Nitrogen	Raw cotton	75	273	343	46.0	-	-	16.3
	OmniBlock coated cotton	219	287	341	57.9	-	-	23.9

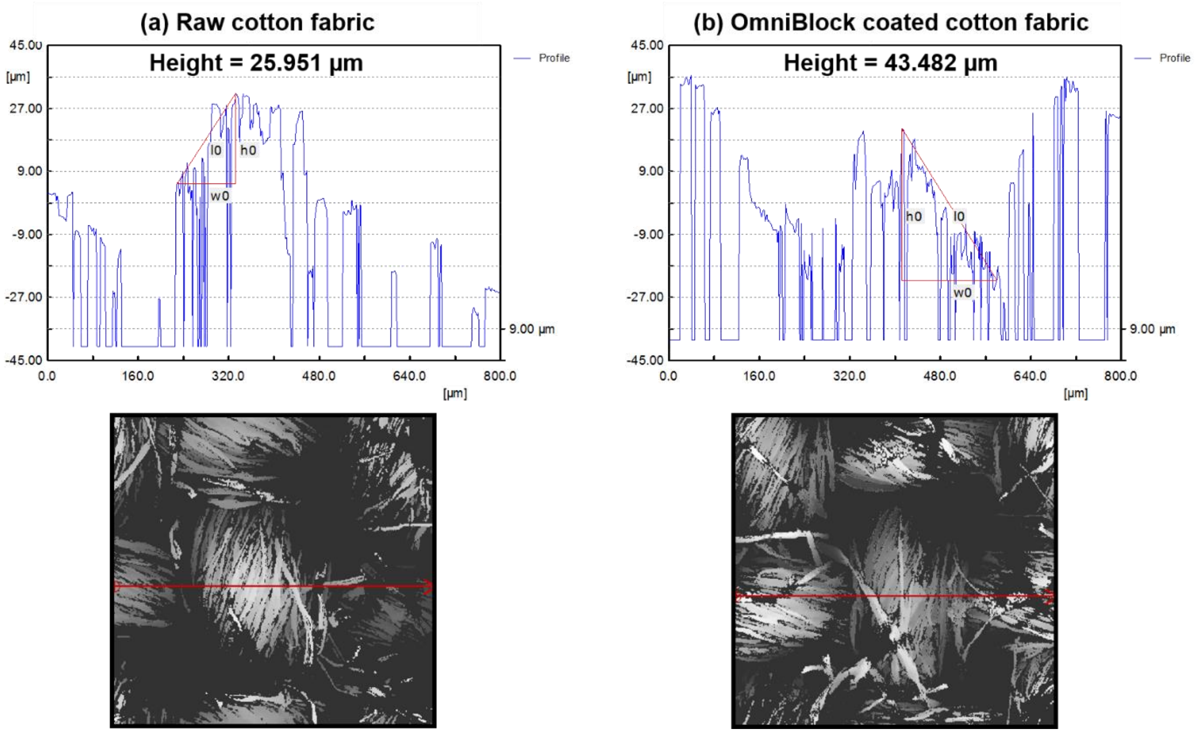

Figure S1. Surfaces for height of small structures of (a) raw cotton fabric and (b) OmniBlock coated cotton fabric.

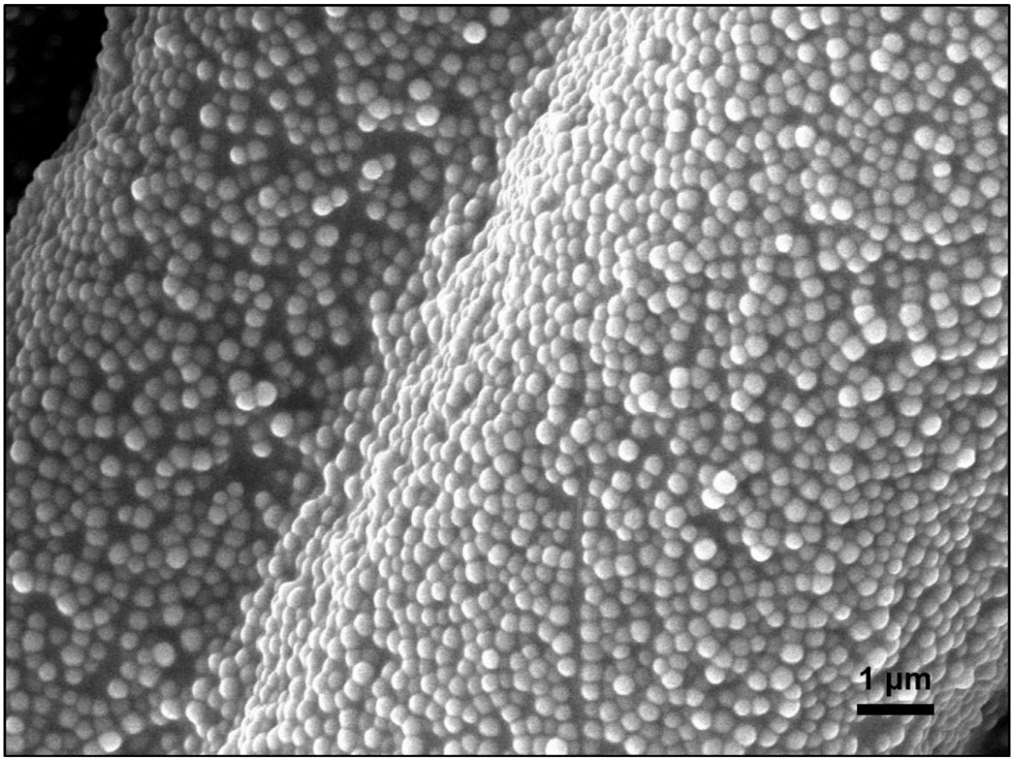

Figure S2. SEM image of OmniBlock coated cotton fabric.

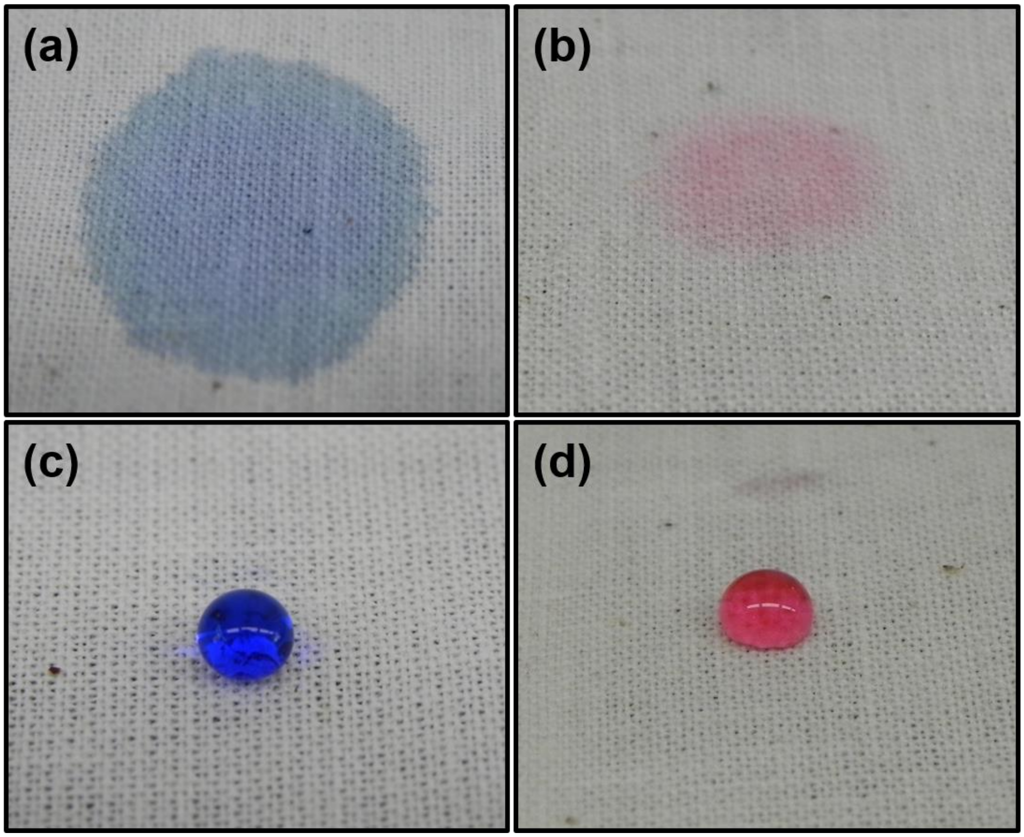

Figure S3. The optical images of (a) water droplet and (b) n-dodecane droplet on Ar plasma treated cotton fabric, (c) water droplet, and (d) n-dodecane droplet on OmniBlock coated cotton fabric

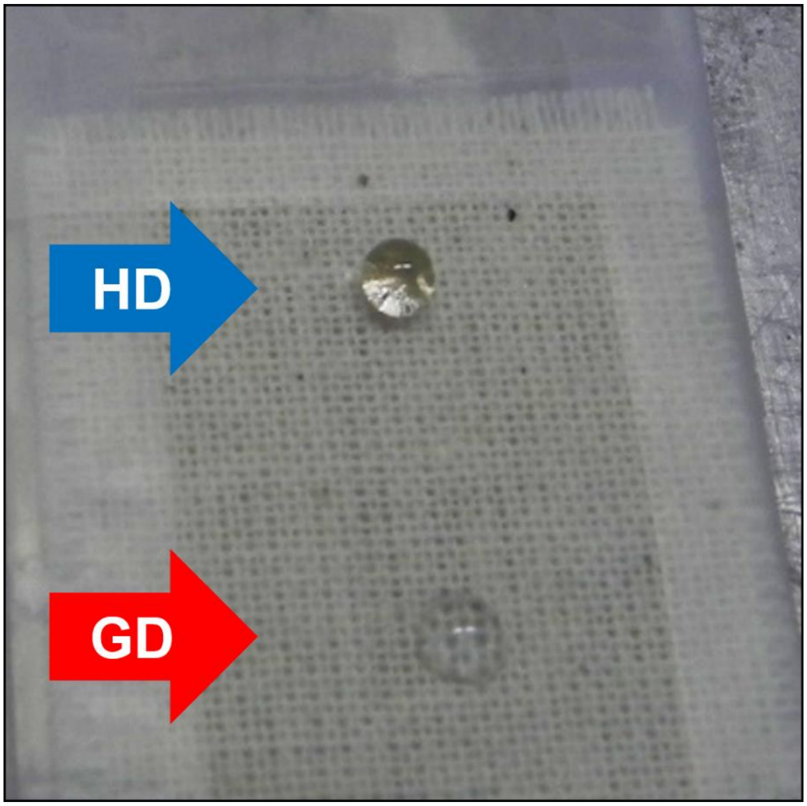

Figure S4. Optical image of HD and GD droplet onto OmniBlock coated cotton.

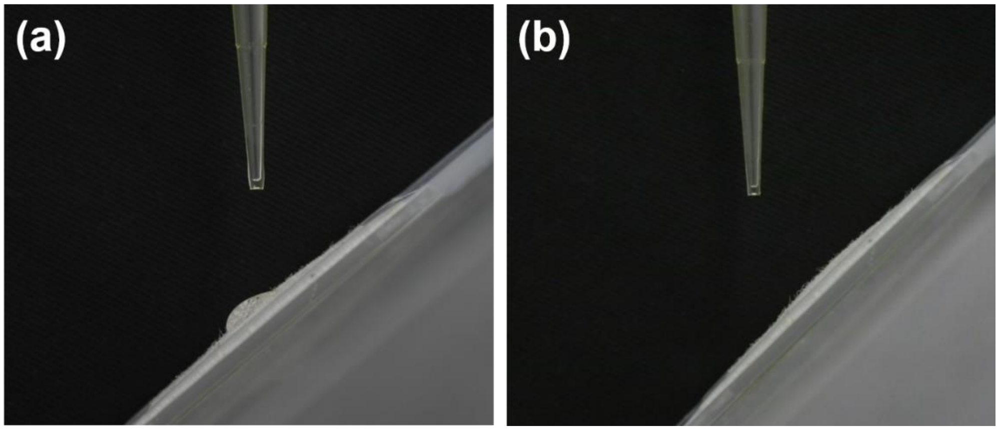

Figure S5. Optical images of shedding angle analysis for n-dodecane on (a) 40°and (b) 45° inclined surface.

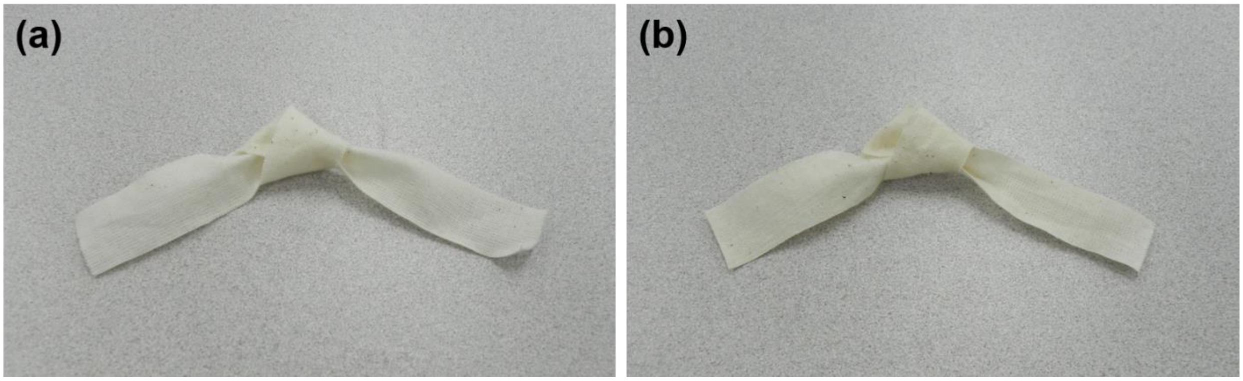

Figure S6. The optical images of knotted (a) raw cotton fabric and (b) OmniBlock coated cotton fabric.
